# Pentraxin 3 Facilitates Shrimp-Allergic Responses in IgE-Activated Mast Cells

**DOI:** 10.1155/2022/8953235

**Published:** 2022-12-07

**Authors:** Jyun-Yi Du, Hong-Yue Lai, Yu-Wei Hsiao, Jhih-Ying Chi, Ju-Ming Wang

**Affiliations:** ^1^Department of Biotechnology and Bioindustry Sciences, College of Bioscience and Biotechnology, National Cheng Kung University, Tainan, Taiwan; ^2^Department of Pharmacology, School of Medicine, College of Medicine, China Medical University, Taichung, Taiwan; ^3^Graduate Institute of Medical Sciences, College of Medicine, Taipei Medical University, Taipei, Taiwan; ^4^Graduate Institute of Medical, College of Medicine, Kaohsiung Medical University, Kaohsiung, Taiwan; ^5^International Research Center for Wound Repair and Regeneration, National Cheng-Kung University, Tainan, Taiwan

## Abstract

**Background:**

Since food avoidance is currently the only way to prevent allergic reactions to shrimp, a better understanding of molecular events in the induction and progression of allergy, including food allergy, is needed for developing strategies to inhibit allergic responses. Pentraxin 3 (PTX3) is rapidly produced directly from inflammatory or damaged tissues and is involved in acute immunoinflammatory responses. However, the role of PTX3 in the development of immediate IgE-mediated shrimp allergy remains unknown.

**Methods:**

Wild-type BALB/c mice were immunized intraperitoneally and were challenged with shrimp extract. Serum IgE and PTX3 levels were analyzed. RBL-2H3 cells were stimulated with either dinitrophenyl (DNP) or serum of shrimp-allergic mice, and markers of degranulation, proinflammatory mediators, and phosphorylation of signal proteins were analyzed. We further examined the effect of PTX3 in shrimp extract-induced allergic responses *in vitro* and *in vivo*.

**Results:**

Mice with shrimp allergy had increased PTX3 levels in the serum and small intestine compared with healthy mice. PTX3 augmented degranulation, the production of proinflammatory mediators, and activation of the Akt and MAPK signaling pathways in mast cells upon DNP stimulation. Furthermore, the expression of transcription factor CCAAT/enhancer-binding protein delta (CEBPD) was elevated in PTX3-mediated mast cell activation. Finally, the PTX3 inhibitor RI37 could attenuate PTX3-induced degranulation, proinflammatory mediator expression, and phosphorylation of the Akt and MAPK signaling.

**Conclusions:**

The results suggested that PTX3 can facilitate allergic responses. Our data provide new insight to demonstrate that PTX3 is a cause of allergic inflammation and that RI37 can serve as a therapeutic agent in shrimp allergy.

## 1. Background

Among all food allergies, allergy to shellfish such as shrimp is particularly common in Asian countries [1, 2]. The main symptoms of a shrimp allergy generally develop within minutes to an hour and vary widely from oral symptoms to life-threatening allergic reactions (such as anaphylaxis). Shrimp allergy is typically lifelong and predominately affects adults; therefore, clarification of the molecular characteristics of shrimp allergy is important for developing drugs and improving clinical management and therapeutic regimens.

The sensing of microbial pathogens and tissue damage through pattern recognition receptors (PRRs) triggers a complex response, including the activation of an acute-phase response, the production of inflammatory cytokines, and leukocyte recruitment to sites of inflammation [3]. In addition, the involvement of PRRs such as Toll-like receptors (TLRs) in food allergy has been reported [4]. Pentraxin 3 (PTX3) is a phylogenetically conserved humoral PRR, which exerts functions in innate immunity, microbial defense, tissue repair and remodeling, and the regulation of inflammation [5]. PTX3 is rapidly produced and released from certain cell types, including macrophages, dendritic cells, neutrophils, endothelial cells, fibroblasts, epithelial cells, adipocytes, and vascular smooth muscle cells, in response to inflammatory cytokines [e.g., tumor necrosis factor-*α* (TNF-*α*) and interleukin-1*β* (IL-1*β*)], TLR agonists, and pathogenic moieties [6, 7].

Immunoglobulin E- (IgE-) mediated shrimp allergy is a leading cause of severe allergic reaction. The high-affinity IgE receptor Fc*ε*RI is highly expressed in mast cells and is required for transmitting signals that induce degranulation and allergic reactions [8]. The crosslinking of Fc*ε*RI with IgE-antigen complexes induces the activation of protein-tyrosine kinases (PTKs) and then PTKs phosphorylate substrates that are involved in the activation of several signaling pathways, including phosphoinositide 3-kinase (PI3K)/Akt, RAS/extracellular signal-regulated kinase (ERK), p38, and JNK [9]. Following the activation of the above signaling pathways, a degranulation reaction and the production of histamine and inflammatory cytokines, including TNF-*α*, IL-4, IL-6, IL-13, and cyclooxygenase-2 (COX-2), are observed in mast cells and basophils [10]. Nevertheless, whether PTX3 is involved in Fc*ε*RI-mediated signaling cascades and responses in mast cells remains an open question.

Physiologically, PTX3 confers host resistance against pathogens [11]. Accumulated evidence suggests that PTX3 is a potential marker associated with disease severity and mortality in diverse human pathological conditions, such as cardiovascular diseases [12], rheumatoid arthritis [13], chronic kidney disease [14], sepsis [15], and several types of cancers [16–18]. In addition, at normal physiological condition, the expression level of CCAAT/enhancer-binding protein delta (CEBPD) is typically low but is rapidly induced by inflammatory factors, such as TNF-*α*, IL-1*β*, IL-6, and COX-2 [19]. Recently, our previous studies have demonstrated that PTX3 is a downstream target of CEBPD and contributes to inflammation-related disorders, such as atherosclerosis [20], Alzheimer's disease [21], rheumatoid arthritis [22], invasion and metastasis of cancer, and drug-resistant cancers [16]. However, very little is known about whether PTX3 and CEBPD can participate in crosstalk between the innate and adaptive immune systems in the initiation or aggravation of shrimp-allergic immune response.

In this study, we found an increase in PTX3 levels in the intestine of shrimp-allergic mice and IgE-induced allergic inflammation. PTX3 can result in exaggerated degranulation, the production of inflammatory mediators, and activation of the Akt and MAPK signaling cascades in IgE-antigen complex- (IgE/Ag-) treated mast cells. In addition, we showed that, upon PTX3 treatment, CEBPD is activated in mast cells and participates in shrimp allergy. Moreover, a PTX3 inhibitor, the RI37 peptides, was applied to prevent the PTX3-induced hypersensitivity reaction and reduce the degranulation of mast cells in *in vitro* and *in vivo* models. Altogether, the results demonstrated the novel role of PTX3 in regulating the IgE-mediated inflammation of DNP- or shrimp-induced allergy in mast cells.

## 2. Methods

### 2.1. Reagents

DMEM and fetal bovine serum (FBS) were purchased from Gibco. ELISA kits for IgE and PTX3 were purchased from Bethyl Laboratories, Inc. and R&D Systems, Inc., respectively. *p*-nitrophenyl-*N*-acetyl-*β*-D-glucosaminide (p-NAG), anti-DNP-IgE antibody, DNP-BSA, 3-(4,5-dimethylthiazol-2-yl)-2,5-diphenyl-tetrazolium bromide (MTT), dimethyl sulfoxide (DMSO), and *o*-phthalaldehyde were obtained from Sigma-Aldrich Co.

### 2.2. Mouse Sensitization and Immunization

Three-four-week-old female BALB/c mice were handled according to the guidelines of our institute (the Guide for Care and Use of Laboratory Animals, National Cheng Kung University). The animal use protocol listed was reviewed and approved by the Institutional Animal Care and Use Committee (IACUC). All mice were maintained on a shrimp-free diet and housed in pathogen-free conditions. To induce shrimp hypersensitivity in mice, sensitization was performed by intraperitoneal injection with 10 mg of shrimp extract plus 1 mg of aluminum hydroxide (Al(OH)_3_) on days 1, 4, 7, 10, 13, 16, 19, and 22, and mice were challenged with 50 mg of shrimp extract on day 30. Mice administered phosphate-buffered saline (PBS) plus adjuvant were included as controls. Blood samples were collected 2 h after the challenge for antibody and PTX3 analysis. *CEBPD*^−/−^ mice (C57BL/6 background) were a kind gift from Sterneck et al. [23]. In *CEBPD*^−/−^ mice, the coding region of *CEBPD* was replaced by a neomycin resistance gene.

### 2.3. Immunohistochemistry (IHC)

Mice were sacrificed by CO_2_ asphyxiation prior to IHC examination. The entire small intestine was dissected longitudinally, cut open along its longitudinal axis, and rinsed briefly in PBS. Intestinal tissues were fixed in 10% formalin in PBS and subsequently embedded in paraffin. Then, 5 *μ*m-thick paraffin sections were cut, and the slides were deparaffinized in xylene and rehydrated in graded alcohol dilutions. The sections were treated with Tris-EDTA buffer (10 mM Tris, 1 mM EDTA, and 0.05% Tween 20; pH 9.0) at 120°C for 10 min in a pressure cooker for antigen retrieval. The sections were incubated with a blocking buffer (2% nonfat dry milk and 0.01% Tween 20 in PBS) for 1 h at room temperature. The slides were then stained using a goat anti-PTX3 antibody (Abcam, 1 : 500 dilution) overnight. The slides were then incubated with polymer and DAB solutions from a Novolink Polymer Detection Systems kit (Leica Biosystems) according to the manufacturer's instructions. The sections were then counterstained with hematoxylin and dehydrated. The sections were mounted in mounting medium (Thermo Scientific) and evaluated with an Olympus BX51 microscope.

### 2.4. Cell Culture

RBL-2H3 cells were obtained from the American Type Culture Collection (ATCC, #CRL-2256) and cultured in DMEM with 10% heat-inactivated FBS at 37°C in a humidified incubator (5% CO_2_ and 95% air).

### 2.5. Degranulation Assay

For the degranulation assay, RBL-2H3 cells were loaded onto a 24-well plate (2 × 10^5^ cells/well) and incubated with 0.2 *μ*g/ml anti-DNP-IgE antibody overnight for cell sensitization. The above cells were washed with 1X PBS, and the cells were stimulated with 1 *μ*g/ml DNP-BSA in PIPES buffer (140 mM NaCl, 5 mM KCl, 0.6 mM MgCl_2_, 1 mM CaCl_2_, 5.5 mM glucose, 0.1% BSA, and 10 mM PIPES; pH 7.4) for 15 min. Histamine content and *β*-hexosaminidase activity in culture supernatants were measured as indicators of degranulation.

Released histamine was evaluated following a previously reported method. First, 100 *μ*l of cell culture supernatant was mixed with 20 *μ*l of 1 M NaOH, and then 25 *μ*l of the reaction solution (1% (*w*/*v*) *o*-phthalaldehyde dissolved in methanol) was immediately added and incubated for 4 min at room temperature. The reaction was terminated by addition of 10 *μ*l of 3 M HCl. The fluorescence intensity was measured at an excitation wavelength 355 nm and emission wavelength 460 nm.

To measure the amount of *β*-hexosaminidase activity released from the cells, cell culture supernatant (25 *μ*l) was mixed with an equal volume of a 5 mM substrate solution (5 mM p-NAG dissolved in 0.2 M sodium citrate buffer, pH 4.5) at 37°C for 1.5 h. The reaction was terminated by adding 200 *μ*l of stop solution (0.1 M Na_2_CO_3_/NaHCO_3_, pH 10.0). The *β*-hexosaminidase activity was determined by measuring the difference in absorbance at 405 nm.

### 2.6. Quantitative PCR (qPCR)

Total RNA was extracted using the TRIsure RNA extraction reagent (Invitrogen). cDNA synthesis was performed with an RT reaction using SuperScript III (Invitrogen). qPCR was conducted using KAPA SYBR FAST qPCR Master Mix (Life Technologies Corporation and Kapa Biosystems Inc.). PCR was conducted using a CFX Connect Real-Time PCR System (Bio-Rad) with the following pairs of specific primers:

*β*-Actin (forward): 5′-GCATTGCTGACAGGATGCAG-3′.

*β*-Actin (reverse): 5′-GTAACAGTCCGCCTAGAAGCA-3′.

*TNF-α* (forward): 5′-GCCTCTTCTCATTCCTGCTTG-3′.

*TNF-α* (reverse): 5′-CTGATGAGAGGGAGGCCATT-3′.

*IL-4* (forward): 5′-‘AGATGGATGTGCCAAACGTCCTCA-3′.

*IL-4* (reverse): 5′-AATATGCGAAGCACCTTGGAAGCC-3′.

*IL-6* (forward): 5′-ACGGCCTTCCCTACTTCACA-3′.

*IL-6* (reverse): 5′-CATTTCCACGATTTCCCAGA-3′.

*IL-13* (forward): 5′-TGAGGAGCTGAGCAACATCACACA-3′.

*IL-13* (reverse): 5′-TGCGGTTACAGAGGCCATGCAATA-3′.

*COX-2* (forward): 5′-CAAGGGAGTCTGGAACATTG-3′.

*COX-2* (reverse): 5′-ACCCAGGTCCTCGCTTATGA-3′.

*CEBPD* (forward): 5 ′-GCCATGTACGACGACGAGAG-3′.

*CEBPD* (reverse): 5 ′-TGTGATTGCTGTTGAAGAGGTC-3′.

Forty cycles were set for quantitative the PCR program, and the relative expression of target genes was calculated following the formula (2^−ΔΔCt^).

### 2.7. Western Blot Analysis

Cells were harvested and lysed with modified RIPA buffer (50 mM Tris-HCl [pH 7.4], 150 mM sodium chloride, 1 mM ethylenediamine tetra-acetic acid, 1% NP40, 0.25% sodium deoxycholate, 1 mM dithiothreitol, 1 mM phenylmethylsulfonyl fluoride, 1 *μ*g/ml aprotinin, and 1 *μ*g/ml leupeptin). Whole-cell lysates were subjected to SDS-PAGE, transferred to a PVDF membrane (Immobilon®-P), and probed with primary antibodies for the target proteins at 4°C overnight. The primary antibodies used included anti-p84 (#GTX70220, GeneTex), anti-p-Akt (#GTX121937, GeneTex), anti-p-ERK [24] Cell Signaling, anti-p-p38 (#9211, Cell Signaling), anti-p-JNK (#4668, Cell Signaling), and anti-CEBPD (#GTX115047, GeneTex). The specific proteins were detected by incubation with a peroxidase-conjugated secondary antibody at room temperature for 1.5 h. Proteins were visualized using an ECL kit (PerkinElmer).

### 2.8. Cell Viability Assay

To assess the viability of mast cells in response to RI37 using an MTT [3-(4,5-dimethylthiazol-2)-2,5-diphenyltetrazolium bromide] assay (Sigma), RBL-2H3 cells were harvested and transferred into 96-well microplates (2 × 10^4^ cells/well) and treated with 300 *μ*g/ml RI37 for 24, 48, and 72 h at 37°C. Then, 20 *μ*l of 5 mg/ml MTT was added, and the cells were incubated for another 3 h at 37°C. The precipitate was dissolved in DMSO, and the absorbance was measured at 570 nm with a microplate reader (iMark™ Microplate Absorbance Reader, Bio-Rad). To assess the proliferation of mast cells using the MTT assay, RBL-2H3 cells were cultured with 300 *μ*g/ml PTX3 for 24, 48, and 72 h.

### 2.9. Short Hairpin RNA (shRNA) Assay

Lentiviruses were produced from Phoenix cells that had been cotransfected with various shRNA expression vectors in combination with pMD2.G and psPAX2. After determining the viral infection efficiency, cells were infected for 48 h with shVoid and shCEBPD lentiviruses, each at a multiplicity of infection (MOI) of 10. The shRNA oligo sequences used in the lentiviral expression vectors were as follows:

shVoid: 5′-CCGGAGTTCAGTTACGATATCATGTCTCGAGACATTCGCGAGTAACTGAACTTTTTT-3′.

shCEBPD: 5′-CCGGGCTGTCGGCTGAGAACGAGAACTCGAGTTCTCGTTCTCAGCCGACAGCTTTTT-3′.

The lentiviral knockdown expression vectors were purchased from the National RNAi Core Facility located at the Genomic Research Center of the Institute of Molecular Biology, Academia Sinica, Taiwan.

### 2.10. Statistical Analysis

All experiments were repeated at least three times, and the data were analyzed for statistical significance using a two-tailed unpaired *t* test (Prism 5 software). One-way ANOVA was performed for multiple comparisons between groups. The data were expressed as the means and standard errors of mean (±SEMs). A statistically significant difference was defined at ^∗^*p* < 0.05, ^∗∗^*p* < 0.01, and ^∗∗∗^*p* < 0.001.

## 3. Results

### 3.1. Elevated Levels of PTX3 in Mice Allergic to Shrimp

**Figure 1:**
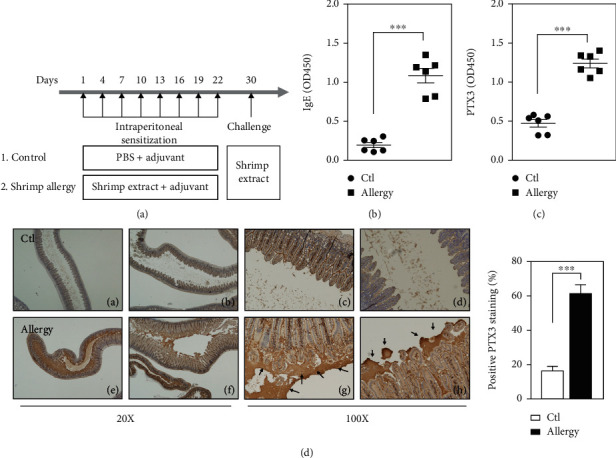
Levels of PTX3 are increased in the serum and small intestine of shrimp-allergic mice. (a) 3-4 weeks old female BALB/c mice (*n* = 6 per group) were intraperitoneally sensitized and challenged with PBS or shrimp extract. Using Al(OH)_3_ as an adjuvant, shrimp allergy mice received shrimp extract plus Al(OH)_3_ in PBS i.p. on days 1, 4, 7, 10, 13, 16, 19, and 22. Blood was collected after the challenge on day 30 for the determination of IgE and PTX3 levels. (b, c) Levels of serological total IgE and PTX3 determined by ELISA. (d) Positive PTX3 immunoreactivity is shown from a representative small intestine of healthy control (a–d) and shrimp allergy (e–h) subjects (from 3 experiments, hematoxylin and PTX3 staining, and magnifications of 20× and 100×). Arrows highlight the outer border of small intestinal villi with increased staining for PTX3. Brown staining indicates PTX3 expression. The quantitative analysis of positive PTX3 staining is shown in the graph.

As mentioned above, the activation of PTX3 has been observed in many inflammation-associated diseases, including asthma [25]. However, the involvement of PTX3 in food allergy remains unknown. To address this issue, a shrimp allergy system was established in BALB/c mice via the intraperitoneal injection of low-dose shrimp extract and adjuvant, Al(OH)_3_, to induce sensitization and then high-dose shrimp extract to induce hypersensitization (Figure 1(a). Allergen sensitization and subsequent challenge are known to induce IgE production in the blood. We found that the level of total IgE in the serum was substantially increased in shrimp-allergic mice compared with that in control mice (Figure 1(b). Next, the ELISA results showed that PTX3 concentrations were significantly higher in the serum on the 30^th^ day of the hypersensitization phase in shrimp extract-challenged mice than in control mice (Figure 1(c). Meanwhile, the results of immunohistochemistry staining showed that positive PTX3 immunoreactivity was detected in all cases in the upper region of the small intestinal villi and lumen after challenge (Figure 1(d). These results show that shrimp allergy induces PTX3 levels higher than those in control mice in the serum and intestinal tracts. Thus, our data imply that PTX3 might play an important role in the allergy to shrimp.

### 3.2. PTX3 Augments Degranulation and the Formation of Proinflammatory Mediators in IgE/Ag-Activated RBL-2H3 Cells

**Figure 2:**
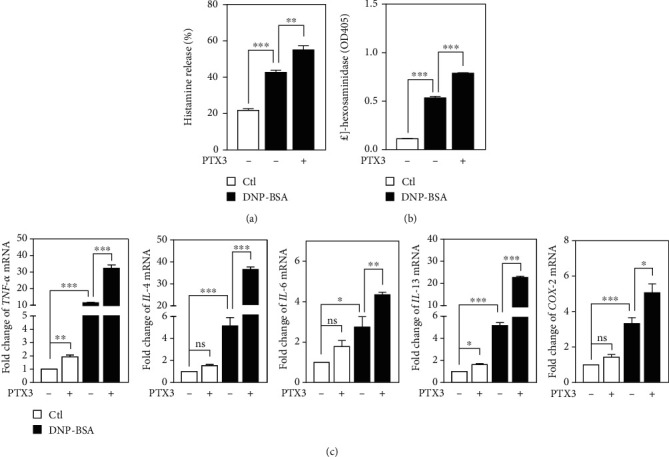
PTX3 augments degranulation and production of proinflammatory mediators in IgE/Ag-activated RBL-2H3 cells. (a, b) RBL-2H3 cells were seeded on a 24-well plate and further incubated with DNP-IgE overnight. IgE-sensitized cells were preincubated with PTX3 (300 ng/ml) for 30 min and then stimulated with DNP-BSA for 15 min. Histamine content and *β*-hexosaminidase activity were determined as described in the Materials and Methods. (c) Effects of PTX3 on the mRNA levels of *TNF-α*, *IL-4*, *IL-6*, *IL-13,* and *COX-2* in IgE/Ag-stimulated RBL-2H3 cells were determined by qPCR. mRNA level of *β-actin* was used as an internal control.

Despite the fact that the clinical symptoms of shellfish allergy are mainly within the gastrointestinal tract, detailed immunological studies in terms of shrimp hypersensitivity are limited. Mast cells and basophils have crucial impacts on the initiation and development of food allergic diseases [26]. The secretion of histamine and *β*-hexosaminidase has been applied as standard biomarkers for determining the degranulation of mast cells. To test whether PTX3 is involved in the enhancement of the degranulation of mast cells, RBL-2H3 cells were first exposed to PTX3 and then stimulated with 2,4-dinitrophenyl (DNP) hapten. Indeed, our results showed that PTX3 could enhance the levels of histamine and *β*-hexosaminidase in IgE/DNP-stimulated RBL-2H3 cells (Figures 2(a) and 2(b). In addition, the increase in de novo-synthesized proinflammatory mediators has been observed in hypersensitization [27, 28]. Therefore, we further examined the effects of PTX3 on the transcriptional activation of proinflammatory cytokines and enzymes associated with the allergic response in RBL-2H3 cells using fluorescent quantitative PCR. The results showed that PTX3 could slightly induce the levels of *TNF-α* and *IL-13* mRNA in RBL-2H3 cells (Figure 2(c)), compare lanes 1 and 2 in each panel). However, PTX3 significantly enhanced the levels of *TNF-α*, *IL-4*, *IL-6*, and *IL-13* but marginally activated *COX-2* mRNAs in IgE/DNP-stimulated RBL-2H3 cells (Figure 2(c), compare lanes 3 and 4 in each panel). These results suggest that PTX3 can facilitate allergic inflammation though promoting degranulation and proinflammatory mediators and contribute, at least in part, to the initiation of the shrimp-allergic response of mast cells.

### 3.3. PTX3 Enhances the Fc*ε*RI-Mediated Signaling Pathways in IgE/Ag-Activated RBL-2H3 Cells

**Figure 3:**
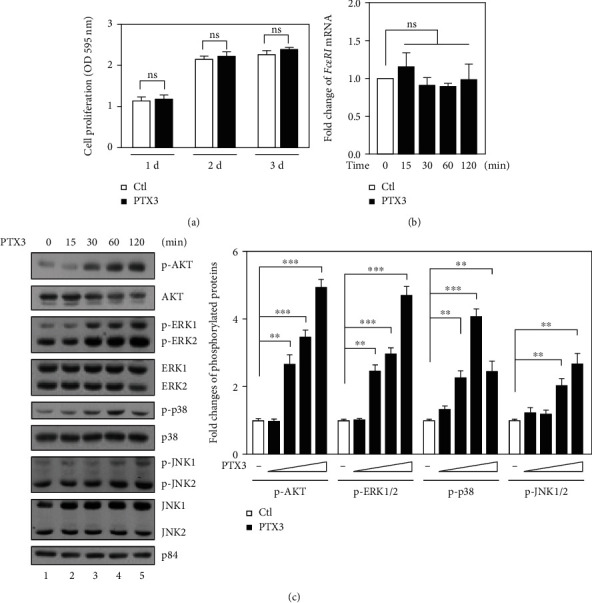
PTX3 contributes to Fc*ε*RI-mediated signaling pathways in RBL-2H3 cells. (a) RBL-2H3 cells were treated with PTX3 (300 ng/ml) for 1, 2, or 3 days. Statistical analysis of cell proliferation was determined by MTT assay. (b) Effects of PTX3 on the mRNA level of *FcεRI* in PTX3-treated RBL-2H3 cells were determined by qPCR. (c) RBL-2H3 cells were incubated with PTX3 for 15, 30, 60, and 120 min. Total lysates were harvested for western blot analysis using antibodies as indicated. The quantitative analysis of phosphorylated proteins is shown in the graph.

PTX3 contributes to cell proliferation in a cell type-dependent manner [29]. Meanwhile, the proliferation of mast cells has also been suggested to be involved in the enhancement of proallergic activity [30]. Therefore, we were interested in examining the involvement of PTX3 in the proliferation of mast cells. The results showed that PTX3 had no effect on the proliferation of RBL-2H3 cells (Figure 3(a)). Furthermore, the increase in and activation of de novo-synthesized Fc*ε*RI and Fc*ε*RI downstream signaling, respectively, have been suggested to be involved and play a critical role in the IgE-mediated allergic reaction in mast cells [31]. We first tested whether PTX3 increases the level of de novo-synthesized *FcεRI* mRNA. The results showed that PTX3 had no effect on increasing *FcεRI* mRNA (Figure 3(b)). We next tested whether PTX3 contributes to the activation of mast cells by enhancing Fc*ε*RI downstream signaling, including Akt, ERK, p38, and JNK [10]. The results showed that PTX3 could enhance the activity of Akt, ERK1/2, p38, and JNK1/2 in IgE-stimulated RBL-2H3 cells (Figure 3(c)). These results suggest that PTX3 is able to augment the activation of Fc*ε*RI-mediated signaling pathways.

### 3.4. PTX3 May Increase Degranulation, the Formation of Proinflammatory Mediators, and the Activation of Fc*ε*RI Cascades in RBL-2H3 Cells Sensitized with Serum from Shrimp-Allergic Mice

**Figure 4:**
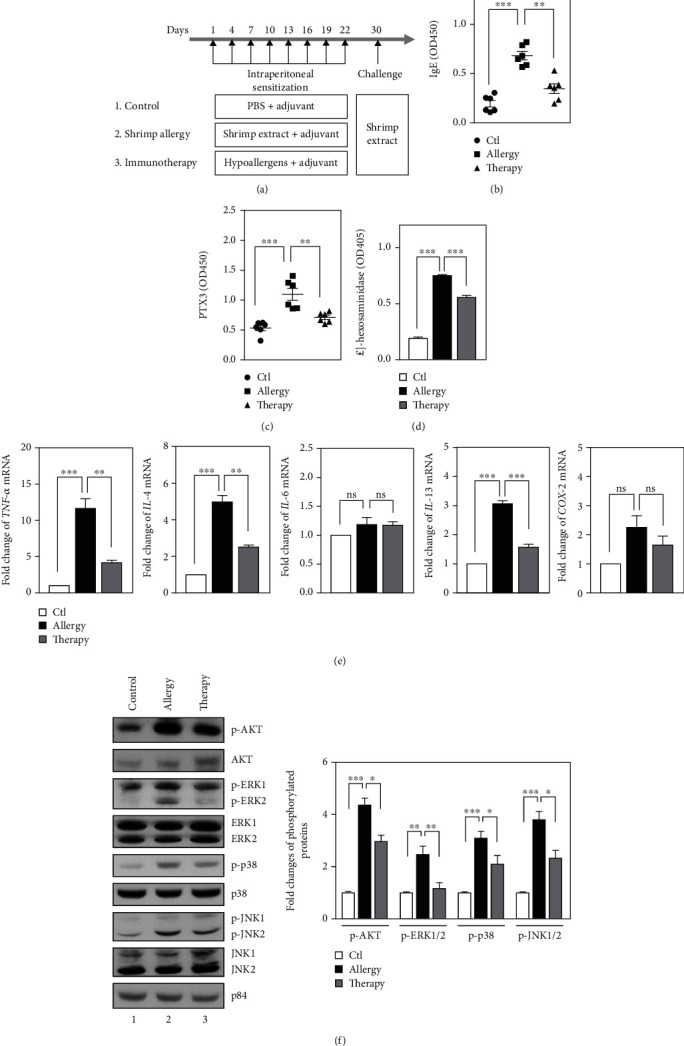
PTX3 increases degranulation, production of inflammatory mediators, and activation of the Fc*ε*RI signaling pathways in RBL-2H3 cells mimicking shrimp allergy. (a) Three-to-four-week-old female BALB/c mice (*n* = 6 per group) were intraperitoneally sensitized with PBS, shrimp extract or recombinant tropomyosin peptides, and challenged by a high dose of shrimp extract. Using Al(OH)_3_ as an adjuvant, shrimp allergy mice received either 10 mg of shrimp extract or 1 mg of tropomyosin peptides in PBS i.p. on days 1, 4, 7, 10, 13, 16, 19, and 22. Blood was collected after the challenge with 50 mg of shrimp extract on day 30. (b, c) Levels of serological total IgE and PTX3 were determined by ELISA. (d) RBL-2H3 cells were incubated with sera from the various groups overnight and further stimulated with shrimp extract for 15 min. *β*-hexosaminidase activity was determined as described in the Materials and Methods. (e) Effects of sera from the various groups as indicated on the mRNA levels of *TNF-α*, *IL-4*, *IL-6*, *IL-13*, and *COX-2* in serum-treated RBL-2H3 cells. (f) Effects of sera from the various groups on Fc*ε*RI signaling pathways in serum-stimulated RBL-2H3 cells. Total lysates were harvested for western blot analysis using antibodies as indicated. The quantitative analysis of phosphorylated proteins is shown in the graph.

Tropomyosin has been reported as a major allergen for shrimp-allergic populations. In addition, pretreatment with tropomyosin peptides that strongly inhibit IgE could attenuate shrimp extract-induced hypersensitization [32]. We further assessed whether the serum of tropomyosin peptide-sensitized mice could attenuate shrimp extract-induced hypersensitization. Following the procedure (Figure 4(a)), we demonstrated a modulation effect of short fragment peptides of tropomyosin (hypoallergens) in the attenuation of shrimp extract-induced hypersensitization (Figure 4(b)). Furthermore, we assessed the levels of PTX3 in the serum of shrimp extract- or tropomyosin peptide-sensitized mice. We found that the levels of PTX3 were positively correlated with the levels of IgE in the serum from shrimp extract- (Spearman′s correlation  = 0.8601 and *p*  = 0.0003) or tropomyosin peptide-sensitized (Spearman′s correlation  = 0.7972 and *p*  = 0.0019) mice (Figure 4(c) and Supplementary Figure 1), suggesting that PTX3 may be involved in IgE-mediated allergic reaction. To develop an *in vitro* model to mimic the immune response of shrimp-induced allergies, the serum from experimental mice was harvested to treat RBL-2H3 cells. Moreover, there were positive correlations among *β*-hexosaminidase activity, expression of several proinflammatory mediators, including *TNF-α*, *IL-4*, and *IL-13*, and the Akt and MAPK signaling pathways in RBL-2H3 cells challenged with the serum from shrimp extract- or tropomyosin peptide-sensitized mice (Figures 4(d)–4(f)). However, *IL-6* and *COX-2* expressions in this *in vitro* system showed no significant change (Figure 4(e)). Taken together, in addition to suggesting that PTX3 could be a diagnostic candidate in shrimp-induced hypersensitization, the results also imply that the inactivation of PTX3 could attenuate shrimp-induced hypersensitization.

### 3.5. CEBPD Is Involved in PTX3-Induced Degranulation and Formation of Proinflammatory Mediators in Mast Cells

**Figure 5:**
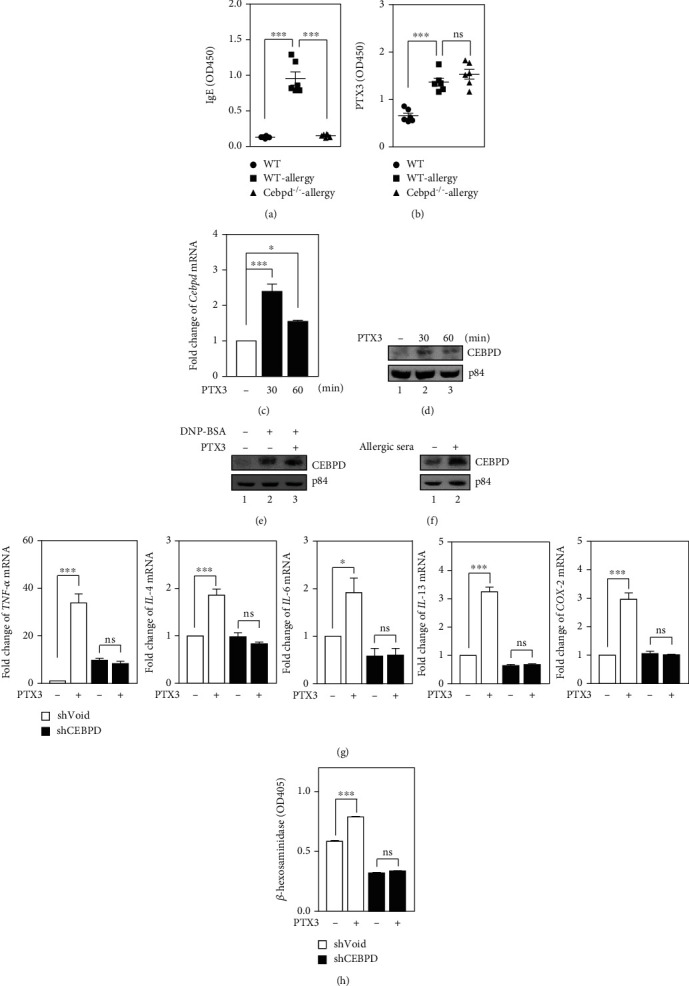
CEBPD is upregulated upon PTX3 treatment and contributes to degranulation and production of inflammatory mediators in mast cells. (a, b) Three-to-four-week-old female C57BL/6 mice (*n* = 6 per group) were intraperitoneally sensitized and challenged with PBS or shrimp extract. Using Al(OH)3 as an adjuvant, shrimp allergy mice received shrimp extract plus Al(OH)3 in PBS i.p. on days 1, 4, 7, 10, 13, 16, 19, and 22. Blood was collected after the challenge on day 30 for the determination of IgE and PTX3 levels. Levels of serological total IgE and PTX3 were determined by ELISA. (c) Effects of PTX3 (300 ng/ml) for 30 and 60 min on the mRNA levels of *CEBPD* in RBL-2H3 cells. (d) RBL-2H3 cells were stimulated with PTX3 (300 ng/ml) for 30 and 60 min. Total lysates were harvested for western blot analysis using antibodies as indicated. (e) IgE-sensitized RBL-2H3 cells were stimulated with DNP-BSA plus PTX3 (300 ng/ml) or not for 30 min. Total lysates were harvested for western blot analysis using antibodies as indicated. (f) Serum-sensitized RBL-2H3 cells were stimulated with shrimp extract for 15 min. Total lysates were harvested for western blot analysis using antibodies as indicated. (g) RBL-2H3 cells were infected with lentiviruses bearing shVoid or shCEBPD and treated with or without PTX3. Total RNA was harvested for qPCR assays. (h) *β*-hexosaminidase activity was determined as described in the Materials and Methods.

Allergen-reactive T helper 2 (Th2) cells have been demonstrated to play a triggering role in allergic inflammation. Interestingly, our previous research has showed that CEBPD can be activated and function not only in M1-like macrophages [20] but also in M2-like macrophages [33], which mirrors Th1 and Th2 responses, respectively. Additionally, our previous studies have also indicated that PTX3 is a downstream target of CEBPD and that the CEBPD/PTX3 axis is involved in some inflammation-related diseases and even in the tumor progression [16, 20, 34]. However, whether CEBPD is reciprocally regulated by PTX3 and contributes to PTX3-induced shrimp hypersensitization remain unexplored. Here, a shrimp allergy system was tested in *Cebpd*-deficient (*Cebpd^−/−^*) mice via the intraperitoneal injection of shrimp extract to induce sensitization and hypersensitization. ELISA analysis revealed that the loss of *Cebpd* in mice suppressed shrimp extract-induced total IgE in the serum (Figure 5(a). Surprisingly, the PTX3 concentration was still significantly high in the serum during the hypersensitization phase in *Cebpd*^−/−^ mice (Figure 5(b)), implying that PTX3 may not be regulated by CEBPD in shrimp allergy. The results prompted us to further examine whether CEBPD is activated by PTX3 and contributes to shrimp allergy. To check the regulation of CEBPD in response to PTX3, IgE/DNP, or allergic serum in mast cells, the expression of CEBPD mRNA and protein were investigated. Upon PTX3 treatment, the CEBPD transcripts and proteins was significantly increased in RBL-2H3 cells (Figures 5(c) and 5(d)). Moreover, RBL-2H3 cells were exposed to PTX3 or not and then stimulated with DNP. The results showed that CEBPD levels were significantly increased in IgE/DNP-stimulated RBL-2H3 cells and were further enhanced under PTX3 treatment (Figure 5(e)). In addition, CEBPD levels were also increased in allergic serum-activated RBL-2H3 cells (Figure 5(f)). The PTX3-induced proinflammatory mediators and *β*-hexosaminidase were attenuated by the knockdown of CEBPD (Figures 5(g) and 5(h)). These results suggest that PTX3-induced degranulation, and the production of proinflammatory mediators is mediated by CEBPD in mast cells.

### 3.6. RI37 Attenuates PTX3- or Shrimp Extract-Induced Allergic Responses

**Figure 6:**
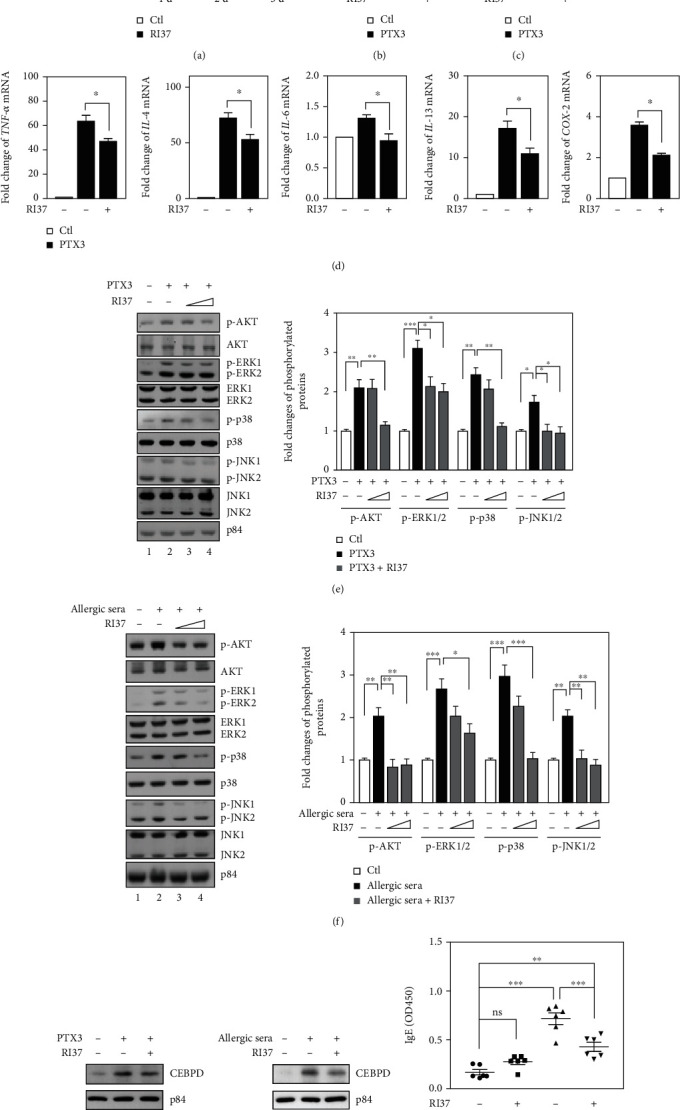
Inhibitory effect of the RI37 peptides on the allergic responses. (a) RBL-2H3 cells were treated with the RI37 peptide (300 ng/ml) for 1, 2, or 3 days. Cell viability was determined by MTT assay. (b, c) RBL-2H3 cells were incubated with DNP-IgE overnight. IgE-sensitized cells were preincubated with RI37 (300 ng/ml) for 30 min and then stimulated with PTX3 (300 ng/ml) plus DNP-BSA for 30 min. Histamine content and *β*-hexosaminidase activity were determined as described in the Materials and Methods. (d) Effects of RI37 on the mRNA levels of *TNF-α*, *IL-4*, *IL-6*, *IL-13,* and *COX-2* in PTX3-treated RBL-2H3 cells were determined by qPCR. The mRNA level of *β-actin* was used as an internal control. (e) RBL-2H3 cells were preincubated with RI37 (150 and 300 ng/ml) for 30 min and then stimulated with PTX3 (300 ng/ml) for 30 min. Total lysates were harvested for western blot analysis using antibodies as indicated. The quantitative analysis of phosphorylated proteins is shown in the graph. (f) Serum-sensitized RBL-2H3 cells were preincubated with RI37 (150 and 300 ng/ml) for 30 min and further stimulated with shrimp extract for 15 min. Total lysates were harvested for western blot analysis using antibodies as indicated. The quantitative analysis of phosphorylated proteins is shown in the graph. (g, h) RBL-2H3 cells were preincubated with RI37 (300 ng/ml) for 30 min and further stimulated with PTX3 or allergic serum for 30 min. Total lysates were harvested for western blot analysis using antibodies as indicated. (i) Three-to-four-week-old female BALB/c mice (*n* = 6 per group) were intraperitoneally sensitized and challenged with PBS or shrimp extract. Using Al(OH)_3_ as an adjuvant, shrimp allergy mice received shrimp extract plus Al(OH)_3_ in PBS i.p. on days 1, 4, 7, 10, 13, 16, 19, and 22. For the RI37 therapy experiment, group 2 and group 4 mice were intraperitoneally immunized three times on days 19, 22, and 30 with 0.1 mg of RI37 adsorbed to PBS plus shrimp extract and shrimp extract alone, respectively. Blood was collected 2 h after the challenge on day 30 for the determination of IgE levels.

In our previous study, the PTX3 inhibitor RI37 peptides (amino acid 200-236) were developed to prevent the PTX3-induced metastasis and invasion of cancer cells [16]. We then tested whether RI37 was able to inhibit PTX3-induced degranulation, proinflammatory mediator formation, and the Akt and MAPK signaling pathways in RBL-2H3 cells. We found that RI37 peptides had no cytotoxicity on RBL-2H3 cells (Figure 6(a)). Pretreatment with RI37 peptides could significantly attenuate PTX3-induced degranulation of IgE/Ag-activated RBL-2H3 cells (Figures 6(b) and 6(c)). Meanwhile, RI37 peptides suppressed PTX3-increased mRNA levels of *TNF-α*, *IL-4*, *IL-6*, *IL-13*, and *COX-2* in IgE/Ag-activated RBL-2H3 cells (Figure 6(d)). Furthermore, RI37 peptides can also inhibit PTX3- or allergic serum-induced activation of Akt and MAPK signaling and CEBPD expression in RBL-2H3 cells (Figures 6(e)–6(h)).

Following successful demonstration of the suppression effect of RI37 peptides in *in vitro* system, the effect of RI37 peptides on shrimp extract-induced allergic responses *in vivo* was examined. The levels of IgE were not significantly different between the negative control and RI37 treatment groups (Figure 6(i), compare group 1 with group 2). Contrary to the elevated levels of IgE in the serum of shrimp-allergic mice, IgE abundance was significantly attenuated in shrimp-allergic mice cotreated with RI37 peptides (Figure 6(i), compare group 3 with group 4). The results suggest that RI37 treatment could be applicable for the amelioration of shrimp allergen-induced inflammation.

## 4. Discussion

The prevalence of food allergies is rising, and the strategies for prevention and treatment are not optimal; therefore, the molecular mechanisms behind these allergic reactions require further examination. PTX3 has been suggested to play an important role in innate immunity and in modulation of the adaptive immune response [35]. Although mast cells are a part of the innate immune system, they can also serve as effectors in the adaptive immune response following IgE/Fc*ε*RI interaction. However, the potential involvement and regulation of PTX3 in mast cells remain obscure. Herein, our study is the first evidence to link and evaluate the involvement of PTX3 in shrimp allergy. Moreover, PTX3 has been suggested to be responsive to proinflammatory cytokines, such as TNF-*α* and IL-1*β*, in epithelial cells. Our current study further demonstrated that PTX3 contributes to the expression of proinflammatory cytokines in mast cells. This finding raises the speculation that PTX3 can reciprocally regulate proinflammatory cytokines in mast cells.

Previous studies showed that surface Fc*ε*RI abundance in mast cells is increased following the increase in serum IgE concentration and that this effect may enhance the ability of mast cells to sense and bind to allergens during allergic responses [36]. In the present study, our observations showed that the level of *FcεRI* mRNA is unchanged in RBL-2H3 cells upon PTX3 treatment (Figure 3(b)). In addition, PTX3 has been suggested to bind to Fc*γ*RIIa and Fc*γ*RIII but not Fc*ε*RI [37]. These observations imply that PTX3 may enhance the activity of Akt, ERK1/2, p38, and JNK1/2 through an Fc*ε*RI-independent pathway. Moreover, accumulating evidence suggests that osteopontin (OPN) enhances IgE-mediated degranulation of mast cells through binding to CD44 [38] and that CD44 can activate the PI3K/Akt and MAPK/ERK pathways in leukemia cells [39]. Interestingly, CD44 may also be involved in PTX3-induced tumorigenesis [16], suggesting that PTX3 could enhance the activity of Akt, ERK1/2, p38, and JNK1/2 through CD44 binding. In addition, the IgE/Fc*ε*RI axis triggers production of multiple cytokines, including TNF-*α*, which could further induce PTX3 production. In this study, our results also showed that, in mast cells, PTX3 can promote the formation of proinflammatory mediators, including IL-4 and IL-13 that have been demonstrated to induce B cell IgE production in a positive-feedback loop [40]. These findings indicate that PTX3 plays a positive role in the IgE-mediated activation of mast cells. Collectively, in mast cells, upon exposure to allergen, PTX3 may be indirectly activated by TNF-*α* and enhance allergic responses through CD44 binding in an Fc*ε*RI-independent manner. However, this speculation needs further investigation.

A recent study reported that CEBPD can induce *PTX3* transcription and that the increase in PTX3 further participated in attenuation of the macrophage-mediated phagocytosis of damaged neurons [34]. Moreover, activation of p38 and JNK has been suggested to be involved in the activation of *CEBPD* transcription [41, 42]. Moreover, CEBPD can be regulated or be reciprocally regulated by TNF-*α*, IL-1*β*, and IL-6 [43]. The pattern of CEBPD is similar to that of PTX3. Here, we demonstrated that PTX3 treatment can increase *TNF-α* and *IL-6* gene expressions as well as the phosphorylation of MAPK signaling in IgE/Ag-activated RBL-2H3 cells. This finding supports PTX3's role in the enhancement of proinflammatory mediator production and implies that CEBPD could be a PTX3 downstream target in shrimp allergy. Indeed, this study identified CEBPD as a novel PTX3 target. Therefore, several factors, including PTX3 and CEBPD, could be regulated in a positive-feedback loop to enhance allergic inflammation.

It is not currently understood why some individuals develop allergic responses to allergenic foods more easily than healthy individuals, but evidence suggests that the tissue microenvironment is important [44]. It is generally believed that factors from the microenvironment can induce phenotypic changes in mast cells. In this study, we demonstrated that increased shrimp allergy severity is associated with an elevated PTX3 level in the serum. We also showed that PTX3 can augment the allergic responses of IgE/Ag-activated RBL-2H3 cells. These results imply that PTX3 could play a predominant role in the microenvironment for mast cell-dependent shrimp allergy. In addition, recent evidence suggests that prophylactic exposure to hypoallergens may be a promising prevention strategy for food allergy [32, 45]. Peptide-based immunotherapy has been proposed as a safe and effective therapeutic strategy. Therefore, a hypoallergen with low/no IgE reactivity is desirable for peptide-based immunotherapy. Tropomyosin is a major allergen in most shrimp species and in other *Crustacea* species, dust mites, and cockroaches [46]. In this study, we demonstrated that the administration of tropomyosin peptides is able to alleviate the allergic responses to shrimp extract in mice with low PTX3 levels (Figure 4(c)). This finding implies that PTX3 may play a detrimental role under several allergic conditions caused by the panallergen tropomyosin.

Currently, the avoidance of food allergens or treatment of food allergies with antihistamines remains the standard of care. Allergy medications such as antihistamines act by the competitive inhibition of histamine at the H1 receptor on the effector cells [47]. However, antihistamines only control the symptoms of food allergy, and these agents are essentially palliative treatments. Moreover, the common side effects of this type of medication include drowsiness and dizziness through its action on the central nervous system. To address the underlying regulation in allergic immune responses and reduce the side effects of antihistamines, the increased understanding of the biology of mast cells gives rise to PTX3, providing a new target for the treatment of shrimp allergy.

## 5. Conclusions

Our data provide the first evidence that PTX3 is activated and contributes to shrimp allergy. The results also provide new insight to suggest that PTX3 participates in the cause of shrimp-allergic inflammation and that the promising application of RI37 peptides as a therapeutic agent.

## Data Availability

The data that support the findings of this study are available from the corresponding author upon reasonable request.

## Abbreviations

Al(OH)_3_: Aluminum hydroxide
CEBPD: CCAAT/enhancer-binding protein delta
DMSO: Dimethyl sulfoxide
DNP: Dinitrophenol
ERK: Extracellular signal-related kinases
FBS: Fetal bovine serum
IHC: Immunohistochemistry
MAPK: Mitogen-activated protein kinase
MTT: 3-(4,5-dimethylthiazol-2-yl)-2,5-diphenyltetrazolium bromide
PBS: Phosphate-buffered saline
PI3K: Phosphoinositide 3-kinase
p-NAG: *P*-nitrophenyl-*N*-acetyl-*β*-D-glucosaminide
PRRs: Pattern recognition receptors
PTKs: Protein-tyrosine kinases
PTX3: Pentraxin 3
TLRs: Toll-like receptors.

## Supplementary Materials

Supplementary Materials Supplementary Figure 1: the correlations between the levels of IgE and PTX3. (A) Comparisons between control and allergic serum groups. (B) Comparisons between allergic serum and tropomyosin peptide-sensitized serum groups.
