# Supplementary figures and images for: Pentraxin 3 Facilitates Shrimp-Allergic Responses in IgE-Activated Mast Cells
Source: J Immunol Res. 2022 Dec 7;2022:8953235. doi: 10.1155/2022/8953235 (PMC9750785; doi:10.1155/2022/8953235)

Sup. Fig. 1

A

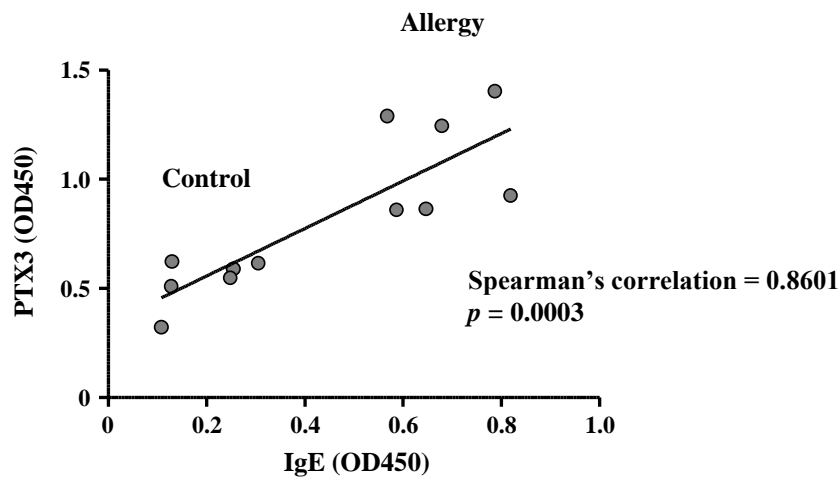

B

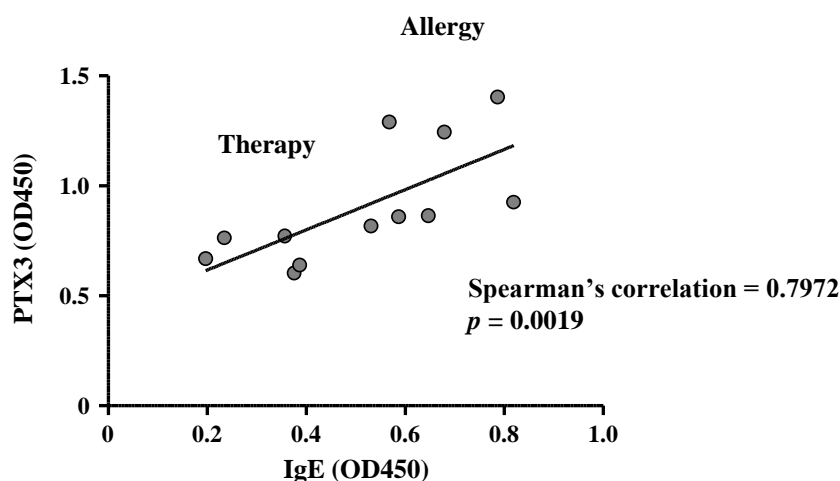

Supplement: Supplementary Materials — Supplementary Figure 1: the correlations between the levels of IgE and PTX3. (A) Comparisons between control and allergic serum groups. (B) Comparisons between allergic serum and tropomyosin peptide-sensitized serum groups. [file 8953235.f1.pdf]
